# Technology-Supported Guidance Model to Support the Development of Critical Thinking Among Undergraduate Nursing Students in Clinical Practice: Protocol of an Exploratory, Flexible Mixed Methods Feasibility Study

**DOI:** 10.2196/31646

**Published:** 2021-10-13

**Authors:** Jaroslav Zlamal, Edith Roth Gjevjon, Mariann Fossum, Simen Alexander Steindal, Andréa Aparecida Gonçalves Nes

**Affiliations:** 1 Lovisenberg Diaconal University College Oslo Norway; 2 Department of Health and Nursing Sciences University of Agder Kristiansand Norway; 3 VID Specialized University Oslo Norway

**Keywords:** critical thinking, guidance model, feasibility, technology, medical education, nursing education, clinical practice

## Abstract

**Background:**

Critical thinking is an essential set of skills in nursing education, and nursing education therefore needs a sharper focus on effective ways to support the development of these skills, especially through the implementation of technological tools in nursing education.

**Objective:**

The aim of this study protocol is to assess the feasibility of a technology-supported guidance model grounded in the metacognition theory for nursing students in clinical practice.

**Methods:**

Both quantitative (research questionnaires) and qualitative (focus group interviews) approaches will be used to collect data for a feasibility study with an exploratory, flexible mixed methods design to test a newly developed intervention in clinical practice.

**Results:**

The intervention development was completed in December 2020. The intervention will be tested in 3 independent nursing homes in Norway.

**Conclusions:**

By determining the feasibility of a technology-supported guidance model for nursing students in clinical practice, the results will provide information on the acceptability of the intervention and the suitability of the outcome measures and data collection strategy. They will also identify the causes of dropout and obstacles to retention and adherence.

**International Registered Report Identifier (IRRID):**

DERR1-10.2196/31646

## Introduction

### Background

Critical thinking is an important outcome of nursing education [1], and clinical practice is essential for its development [2]. In clinical practice, a nurse preceptor serves as a tutor or mentor to guide nursing students toward the acquisition of necessary skills [3].

Nursing students may experience challenges and difficulties in their clinical practicum, such as not knowing who the main nurse preceptor responsible for guidance is, receiving limited guidance, experiencing a change of nurse preceptor, or having a poor relationship with the nurse preceptor [4]. Likewise, nurse preceptors may lack the resources, experience, and training in guiding nursing students [5-7]. These challenges in guiding nursing students in clinical practice may negatively influence their development of critical thinking [8].

The introduction of technological tools in nursing education has opened new possibilities for addressing these challenges and improving outcomes related to critical thinking [9], but only a few studies have examined the effectiveness of technological tools in supporting the development of critical thinking skills in nursing students. Strandell-Laine developed a technological intervention to improve cooperation between nursing students and nurse educators to improve self-efficacy and nursing competence; the intervention was not significantly effective in improving individual outcomes, but it strengthened communication between students and nurse educators [10]. Mettiäinen developed a technology-based app for feedback and assessment in the clinical guidance of nursing students [11]. In a pilot study, Mettiäinen et al [11] found that nursing students had positive attitudes toward the use of technological tools (eg, apps) during their guidance in clinical practice, and she concluded that such apps are a viable option for the guidance of nursing students in clinical practice.

Owing to the importance of critical thinking in nursing education, interventions that support critical thinking and its development are needed. This study provides a protocol for a feasibility study, which is one stage of a complex intervention [12]. The feasibility study is a part of the main study, *Technology-Supported Guidance to Increase Flexibility, Quality, and Efficiency in the Clinical Practicum of Nursing Education*, conducted at Lovisenberg Diaconal University College (LDUC), Oslo, Norway. The main study included a mixed methods systematic review, feasibility study, randomized controlled trial (RCT), and follow-up study. Protocols for the systematic review of mixed methods [13] and RCTs [14] have already been published.

### Study Aim

The overall aim of this study is to explore the feasibility of a technology-supported guidance model for nursing students in clinical practice.

### Objectives

The purpose of this study is to assess the feasibility and acceptability of a newly developed technology-supported guidance model in clinical practice among nursing students, nurse preceptors, and nurse educators; assess the feasibility and suitability of the primary and secondary outcome measures; assess the recruitment strategy; assess the data collection strategy; and identify potential causes of dropout and hindrances to participant recruitment, retention, intervention fidelity, and adherence to the intervention.

### Research Questions

How feasible and acceptable is the newly developed technology-supported guidance model and the overall intervention among nursing students, nurse preceptors, and nurse educators? Are the outcome measures feasible and suitable for an RCT? How feasible is the chosen data collection strategy? How suitable is the participant recruitment strategy? What causes dropout and what hindrances can occur in relation to recruitment, retention, intervention fidelity, and adherence? How can these hindrances be minimized?

## Methods

### Overview

According to Giangregorio and Thabane [15], there is no universal agreement on the definitions of feasibility and pilot studies. Some definitions may overlap, whereas others distinctively differ in their understanding of feasibility and pilot studies. The Medical Research Council Framework for Complex Interventions does not make a clear distinction [16], whereas the National Institute of Health Research in the United Kingdom defines feasibility studies as those that are conducted in the early stages of the research process, before a pilot study, and aim to answer specific questions related to potentially conducting a given intervention research. Pilot studies are then defined as small versions of a main study that aim to determine whether all the components of the main study work together [12].

This study adopts the understanding of feasibility studies outlined by the National Institute of Health Research and focuses on the feasibility stage of intervention research, aiming to inform an RCT. The protocol has been written according to the Standard Protocol Item: Recommendations for Interventional Trials (SPIRIT) checklist [17], Medical Research Council Framework for Complex Interventions [16], and Template for Intervention Description and Replication (TIDieR) [18].

Feasibility studies have, by their nature, an exploratory design that aims to justify a full-scale effectiveness study [19]. In this study, we plan a flexible, convergent, and mixed methods exploratory design. A flexible exploratory design allows for changes during the course of the study, which can inform adjustments to the intervention and final intervention design [19], whereas a convergent mixed methods design allows the comparison of quantitative and qualitative data to confirm or disprove the findings of each approach [20]. Quantitative data will be collected from questionnaires and from the use data of the Technology-Optimized Practice Process in Nursing (TOPP-N) app [21]. Qualitative data will be collected from focus group interviews with participating nursing students, nurse preceptors, and nurse educators. Quantitative data will be analyzed using descriptive statistical methods [22]. We will calculate means, medians, SDs, skewness, and kurtosis [23,24] and report sample sizes and sample demographics [24], such as ages of participants, last completed education, and previous working experience in health care. A thematic analysis approach will be applied to qualitative data. The data will be coded, and the codes will be grouped into themes [25]. The quantitative and qualitative data will be integrated in a side-by-side comparison and interpreted in the Discussion section. Qualitative data will be reported and interpreted first and then compared with the quantitative findings to answer the research questions of the feasibility study [20].

### Study Setting

The feasibility study will be conducted at 3 nursing homes, 1 in the county of Oslo, Norway, and 2 in the county of Kristiansand, Norway. The institutions were chosen based on previous cooperation and agreement in developing or testing the intervention.

### Eligibility Criteria

The study will use a consecutive sampling strategy. Eligible participants include first-year undergraduate nursing students at LDUC and the University of Agder (UiA), nurse preceptors (registered nurses) and nurse educators at the participating institutions, nursing students in clinical practice, nurse preceptors and nurse educators guiding nursing students in clinical practice, and participants who are willing to provide signed informed consent.

### Intervention Description

#### Intervention Name

The name of the intervention is Technology-Supported Guidance Model (TSGM).

#### Goal of the Elements Essential for the Intervention

The main element of the TSGM is the TOPP-N app [21], which helps students identify their need for guidance and stimulates reflection on their learning goal and what has been learned through their completion of electronic reports (e-reports).

Nurse preceptors and nurse educators can follow up on the progress of students and tailor their guidance based on their needs.

Nurse educators follow the students’ guidance and intervene as necessary when automatically prompted by the guidance app.

A digital version of the Assessment of Clinical Education (AssCE) [26] mediates the summative evaluation of student performance during clinical practice with either in-person or virtual meetings.

#### Materials

Materials include the TOPP-N app [21] with a digital AssCE [27] module, accessible from mobile phones, tablets (Apple [iOS] or Android operating system), and web browsers (all standard browsers are supported).

The app can be accessed from a web browser [21] or from Apple or Android systems downloadable from the Apple Store and Google Play, respectively. The informational materials in the training include flyers, posters, instructional videos, a Facebook group, and formal and informal meetings (Multimedia Appendix 1).

Videos can be found on the web [28].

#### Procedures

**Figure 1:**
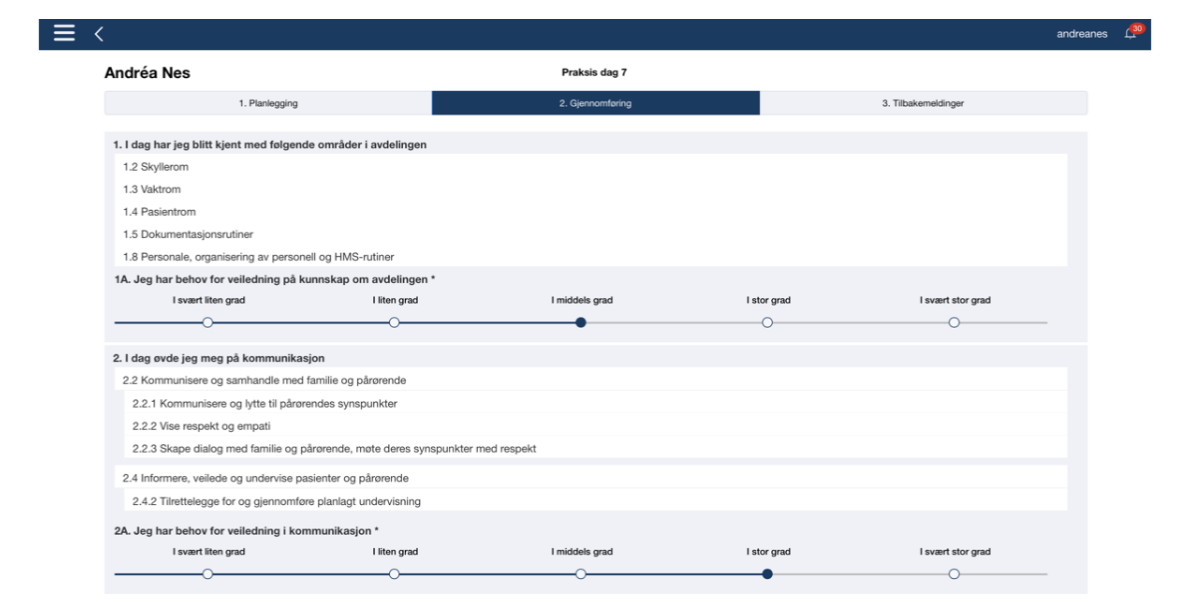
Screenshot of the Technology Optimized Practice Process in Nursing app.

Nursing students use the TOPP-N app (Figure 1) [21] daily and must complete e-reports before and after their shift in clinical practice. The e-reports comprise checklists built on AssCE [27], each of which is accompanied by a scale on which the students indicate their need for guidance in specific learning activities. The checklist offers the possibility of further written elaboration.

Nurse preceptors are required to give feedback on the daily performance of students and on completed e-reports through the TOPP-N app [21]. Feedback is given every day after the students have completed their reports.

Nurse educators follow the students’ progress through the TOPP-N app [21] and intervene as necessary, when automatically prompted by the guidance app.

Summative assessment is done in the app with the help of the digital AssCE [27] in weeks 3 to 4 and 6 to 8 of the students’ clinical practice. The summative assessment is conducted as an individual meeting (physical or virtual) in which students, nurse preceptors, and nurse educators participate.

#### Delivery of Intervention

The intervention is delivered digitally by the TOPP-N app [21]. Daily guidance is delivered by nurse preceptors and, when necessary, by nurse educators. Summative assessment is delivered by nurse preceptors and nurse educators in collaboration with nursing students.

#### Modes, Place, and Frequency of Intervention Delivery

The intervention is delivered digitally through the TOPP-N app [21] and in virtual and face-to-face meetings between nursing students, nurse preceptors, and nurse educators, in 1 nursing home in Oslo, Norway, and 2 nursing homes in Kristiansand, Norway. It is delivered daily during 6 to 8 weeks of clinical practice.

#### Intervention Monitoring

The intervention is monitored digitally by oversight of the participants’ activities and their interactions in the TOPP-N app [21].

#### Criteria for Modifying or Discontinuing an Intervention

Chan et al [17] highlighted the necessity of carefully considering when an intervention should be modified or stopped, and progression criteria are necessary elements of feasibility and pilot studies to evaluate whether a full-scale trial is viable [26]. Avery et al [29] proposed a traffic light system for progression criteria: green (go, indicates the criteria are met); amber (amend, indicates a need for change and adjustment); and red (stop, indicates that one should not move to a larger trial). Following Avery et al [29], the progression criteria are as follows: green (intervention proceeds as planned, and no problems are discovered), amber (problems are discovered and appropriate remedies are devised, and the intervention proceeds with close monitoring), and red (problems cannot be amended, and the intervention does not continue).

#### Adherence to the Intervention Protocol

Adherence describes the behavior of participants that aligns with the intervention and has been assigned to the participants [17]. Poor adherence may complicate statistical analysis, reduce the statistical power of the study, and result in underestimation of the efficacy of the intervention [30]. In this study, the guidance app has a built-in system that reminds participants to fill out e-reports and complete other required tasks.

#### Concomitant Activities and Other Activities Outside of Intervention

No limitations are imposed on the participants in relation to concomitant activities or other activities outside of the intervention.

### Outcomes

The primary outcome is critical thinking. The secondary outcomes are self-efficacy, clinical learning environment, metacognition and self-regulation, technology acceptance, and competence of mentors. Table 1 provides a detailed overview of these outcomes.

**Table 1:** Outcomes.

Outcomes		Definition
**Primary outcome**		
	Critical thinking	Purposeful and self-regulatory judgment resulting in interpretation, analysis, evaluation, and inference efficacy [31].
**Secondary outcomes**		
	Self-efficacy	Self-perceived ability to perform a task in a competent and effective manner [32,33].
	Satisfaction with the clinical learning environment	A clinical learning environment that provides students with professional development and is a foundation for a supervisory relationship [34].
	Technology acceptance	Acceptance or rejection of the use of new technology by users, with a focus on users’ perceptions, attitudes, and intentions in the use of new technology [35].
	Use of metacognitive processes	Use of metacognitive processes in clinical practice [36].
	Mentors’ competence	Level of competencies of mentors in clinical practice [37].

### Participant Timeline

**Figure 2:**
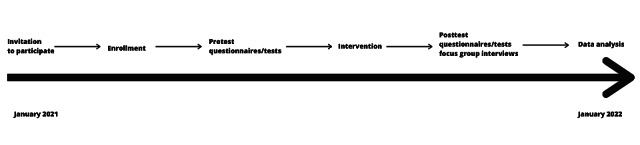
Participant timeline.

The participant timeline is shown in Figure 2.

#### Sample Size

Traditional sample size calculations are not suitable for feasibility studies, as their aim is not hypothesis testing [38,39], yet a feasibility study requires a proper sample size justification [39], especially in relation to its objectives [40]. Lancaster et al [40] have proposed 30 participants as *the rule of thumb*, but recommendations vary from 12 to 50 participants [41]. The current estimate for a sufficient number of participants, as described by Billingham et al [41], is between 12 and 50. For this study, we have decided to recruit a total of 32 nursing students (16 from LDUC and 16 from UiA) and 27 nurse preceptors (13 from LDUC and 14 from UiA).

#### Recruitment

The participants will be recruited from first-year undergraduate nursing students at LDUC, Oslo, Norway, and UiA Kristiansand, Norway. Drawing on the recommendations for recruitment in health research, the recruitment process will provide sufficient information about the overall study in meetings with the target group and will highlight its aim and benefits for participants [42]. To boost recruitment, we intend to maintain a prominent presence on social media.

### Data Collection Methods

Data for the primary outcome will be collected using the Norwegian version of the Health Science Reasoning Test [43]. Data for the secondary outcomes will be collected using the Norwegian version of the Self-Efficacy in Clinical Performance [44], Clinical Learning Environment, Supervision and Nurse Teacher [45,46], Technology Acceptance Model 3 [47], Mentors Competence Instrument [37], and Self-Regulation and Metacognition in Clinical Practice instruments (self-created questionnaire for the purposes of this study).

In addition, data will be gathered from the TOPP-N app [21], and questionnaires will solicit self-reported sociodemographic data and evaluations of participation in the feasibility study. All data collection instruments will be administered digitally.

Data will also be collected through focus group interviews with nursing students, nurse preceptors, and nurse educators. The interviews will be conducted separately for each group using an interview guide and will last 60 minutes. One researcher will be the interviewer and the other a moderator. All focus groups will be conducted digitally using Zoom (Zoom Video Communications, Inc) videoconferencing version 5.6.5 [48]. Video from the interviews will be recorded, but only the sound file will be stored, and the video will be deleted at the end of the focus group interviews. Table 2 provides an overview of the data collection instruments. Textbox 1 presents the planned focus group interview topics.

**Table 2:** Overview of data collection instruments.

Measuring instrument	Characteristics of the instrument	Internal validity
HSRT^a^	Multiple-choice test, 38 questions Measurement of overall level of critical thinking Measurement of detailed scores of analysis, interpretation, inference, evaluation, explanation, induction, deduction, and numeracy	Cronbach α of .76 for the overall instrument [49]
SECP^b^	Measurement of self-efficacy on 37 items in 4 subscales: assessment, diagnosis and planning, implementation, and evaluation	Cronbach α for each item ranging from .90 to .92 [44]
CLES+T2^c^	Measurement of satisfaction with the clinical learning environment on 45 items in three major themes: learning environment, supervisory relationship, and role of the nurse teacher	Cronbach α for each item ranging from .81 to .98 [45,46]
TAM 3^d^	Measurement of acceptance of new technology on 37 items	Cronbach α for each item ranging from .77 to .87 [50]
SMCP^e^	Measurement of level of use of self-regulation and metacognitive processes; measured on 11 items	Data not available
Sociodemographic data	Year of birth, sex, last completed education, length of employment in health care with direct patient contact	Data not available
Evaluation of the feasibility study	Evaluation of participation in the feasibility study	Data not available

^a^HSRT: Health Sciences Reasoning Test.

^b^SECP: Self-Efficacy in Clinical Performance.

^c^CLES+T2: Clinical Learning Environment, Supervision and Nurse Teacher.

^d^TAM 3: Technology Acceptance Model 3.

^e^SMCP: Self-Regulation and Metacognition in Clinical Practice.

**Planned topics in focus group interviews.**

**Nursing students**

- Platform from which Technology Optimized Practice Process in Nursing (TOPP-N) has been used
- Use of TOPP-N
- Contribution of TOPP-N to performance of students in clinical practice
- Contribution of TOPP-N in receiving guidance from nurse preceptors
- Future needs for support when using TOPP-N
- Experience with filling out questionnaires and taking the critical thinking test
- Recruitment to intervention

**Nurse preceptors**

- Platform from which TOPP-N has been used
- Use of TOPP-N
- Contribution of TOPP-N in student guidance
- Comparison of using TOPP-N in guidance of students with earlier guidance without TOPP-N
- Future features and needs in TOPP-N
- Contribution to research which includes filling out questionnaire and time use
- Recruitment to intervention

**Nurse educators**

- Platform from which TOPP-N has been used
- Use of TOPP-N
- Contribution of TOPP-N in student guidance
- Comparison of using TOPP-N in guidance of students with earlier guidance without TOPP-N
- Future features and needs in TOPP-N
- Contribution to research which includes filling out questionnaire and time use
- Recruitment to intervention

### Data Retention

To maintain interest in the study, announcements will be placed on the learning management platform Canvas (Instructure, Inc) [51], and nurse educators will closely communicate with students to support them as necessary. A dedicated support person will also be available to the participants.

### Data Management

Participants’ personal information and sociodemographic data and data from Self-Efficacy in Clinical Performance, Clinical Learning Environment, Supervision and Nurse Teacher, Technology Acceptance Model 3, and Self-Regulation and Metacognition in Clinical Practice will be collected by the Questback Management System (Questback Group AS) [52] and the results stored in the Questback system.

The Health Sciences Reasoning Test is conducted through the Insight Assessment testing system [43], a division of California Academic Press. The anonymous results are stored in the Insight Assessment system. A backup of personnel data and the results of the critical thinking test and other questionnaires will be stored on a Kingston DataTraveller 2000 USB stick with AES 256-bit encryption.

### Methods of Analysis

For quantitative analysis, we will use SPSS, version 26 (IBM Corporation) [53]. For qualitative analysis, we will use MAXQDA Analytics Pro 2020 version 9 (VERBI GmbH) [54].

### Program Theory

The intervention is theoretically based on the concept of metacognition, which is regarded as a higher-order thinking skill and describes the cognitive process of *thinking about one’s own thinking* [55]. It is the ability to be aware of, reflect on, and use strategies during cognitive tasks. People who demonstrate high metacognitive abilities tend to be more focused, thoughtful, and strategic in making decisions and solving problems [56]. Thus, they view their own competence as a dynamic and formable entity, which motivates them to learn from previous knowledge and experiences and seek new solutions. Metacognition is often framed as a highly cognitive skill; however, there is a high correlation between metacognition and self-regulation, which means that metacognition also depends on motivational elements, such as goal setting, determination, and attention control [55,57].

Metacognition is used as a theoretical framework for TSGM because research shows a close interrelationship between metacognition and critical thinking [58]. Thus, we assume that, if the guidance app supports metacognitive skills of students in clinical practice, it will also have a positive effect on their critical thinking skills. The interrelationship between the two concepts can be traced to the importance of self-monitoring and self-reflection in understanding information and thinking through discussions regarding learning and problem-solving [59].

More specifically, the intervention will build on the principles of the metacognitive cycle, which comprises three main phases that together make up a metacognitive process. The first phase is planning and setting goals. Goal setting is an important part of metacognition, as it prepares students to be attentive, aware, and focused on the learning objectives and strategies they will use in pursuing them.

Experts often take more time than novices do in preparing to solve a problem [60]. As metacognitive masters in their domain, they show the wisdom of making considerable preparation before entering the second phase of the cycle.

In the second phase, the planned strategies are applied in the situation. Here, it is important not to be constrained by the planned actions and to maintain self-awareness and higher-order thinking during the activity so that ongoing decisions can be adapted to situational demands.

The third phase occurs after the situation has played out. Now, it is important to engage in critical self-evaluation and reflect on how the applied strategies dealt with situational demands and contributed to achieving the goals established in the first phase. Furthermore, self-evaluation will provide invaluable information when once again entering the first phase and planning new goals and strategies. An important part of this process is the feedback from nurse preceptors, which further stimulates critical self-evaluation and reflection.

**Figure 3:**
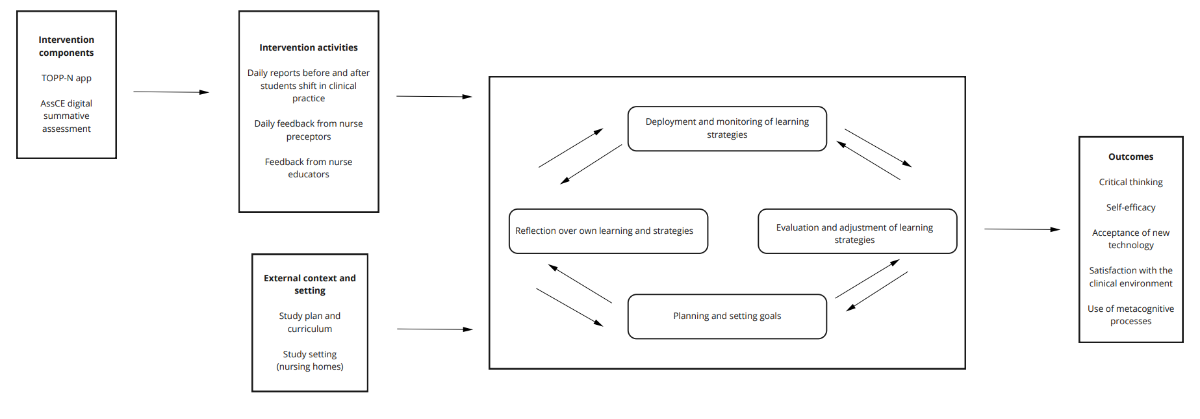
The program theory. AssCE: Assessment of Clinical Education; TOPP-N: Technology-Optimized Practice Process in Nursing.

These phases may be further influenced by factors such as task constraints, beliefs about learning, awareness of one’s own strengths and weaknesses, and individual motivation. Research also shows that metacognition, similar to most cognitive abilities, is not a wholly general ability [55], meaning that advanced metacognitive abilities are not necessarily transferred from one domain to another and that they should be practiced in the relevant context. Figure 3 is a diagrammatic representation of the program theory.

### Research Ethics Approval

The study was approved on December 21, 2020, by the Norwegian Centre for Research Data (reference number: 338576).

### Changes in Protocol

Protocol modifications will be communicated in subsequent publications in research journals.

### Ethics

Each participant signs a written informed consent form. Informed consent is obtained digitally through Questback [52]. The students are thoroughly informed (both verbally and in writing) that participation or nonparticipation in the research project will not affect their study progression or the evaluation of their performance. None of the researchers participating in this research study was involved in any form of formal teaching, evaluation, or student follow-up. This is important in preventing potential conflicts of interests [17].

### Confidentiality of Information

On agreeing to participate, each participant receives a numerical code, which is their identifiable information. The numerical codes will be kept separately from the actual list of the participants.

### Dissemination Policy

According to Craig et al [16], results should be disseminated actively and targeted in a way that makes them easily understandable and accessible. The research findings will be disseminated by publishing research articles in open-access research journals. In addition, the research team of the study will ensure a strong presence on social media and promote the publication of relevant articles in the daily press, where the findings and news about the research results will be disseminated in a manner easily understandable to a wider audience. Conference participation is also part of the dissemination strategy of the study.

## Results

The feasibility study was completed in March of 2021. Quantitative data (from questionnaires) were collected at baseline, before the feasibility study began, and after its completion. We collected qualitative data (focus group interviews) in April of 2021. Table 3 provides a detailed timeline of the further stages of the analysis. This study is expected to conclude in January 2022.

**Table 3:** Detailed timeline of further stages of analysis.

Data analysis		Timeline
**Quantitative data**		
	Calculation of means, medians, SDs, skewness, kurtosis	August 2021
	Reporting of sample sizes and sample demographics	August 2021
**Qualitative data**		
	Transcription of focus group interviews	June to July 2021
	Analysis of focus group interviews	August to October 2021
	Integration of qualitative and quantitative data	November to January 2022

## Discussion

### General

Critical thinking is an essential skill set in nursing [61], and previous research underscores the need for more quantitative approaches to critically evaluate how critical thinking skills are developed, especially among nursing students in a clinical setting [62].

### Significance of Results

The feasibility study offers the advantage of testing and fine-tuning certain parts of the main study [63].

### Limitations

A limitation of this study is that the intervention has many complex parts that require close monitoring and follow-up, and the feasibility study runs alongside the control group arm of the trial. Consequently, it may not be possible to use all the results to fine-tune the intervention and the trial (eg, the choice of outcome or data collection instruments). The decision to run the feasibility study alongside the control group arm of the trial was made for practical reasons related to how the curriculum and clinical practice are organized, particularly in the context of the influence of the COVID-19 pandemic on the operation of clinical practice.

### Conclusions

The results will determine the acceptability and suitability of the intervention, as well as the information collection strategy and outcome measures for a technology-supported guidance model for nursing students in clinical practice, as well as dropout causes, adherence challenges, and retention.

## Appendix

Multimedia Appendix 1 Poster used in participant recruitment.

## Abbreviations

AssCE: Assessment of Clinical Education
LDUC: Lovisenberg Diaconal University College
TIDieR: Template for Intervention Description and Replication
TOPP-N: Technology-Optimized Practice Process in Nursing
TSGM: Technology-Supported Guidance Model
UiA: University of Agder
