# Supplementary material for: Technology-Supported Guidance Model to Support the Development of Critical Thinking Among Undergraduate Nursing Students in Clinical Practice: Protocol of an Exploratory, Flexible Mixed Methods Feasibility Study
Source: JMIR Res Protoc. 2021 Oct 13;10(10):e31646. doi: 10.2196/31646 (PMC8552102; doi:10.2196/31646)
Supplement: Multimedia Appendix 1 [file resprot_v10i10e31646_app1.pdf]

Til deg som er praksisveileder!

Opplever du at det er utfordrende å veilede sykepleierstudenter?
Strekker ikke tiden til når du veileder studenter?
Vil du øke din veiledningskompetanse?

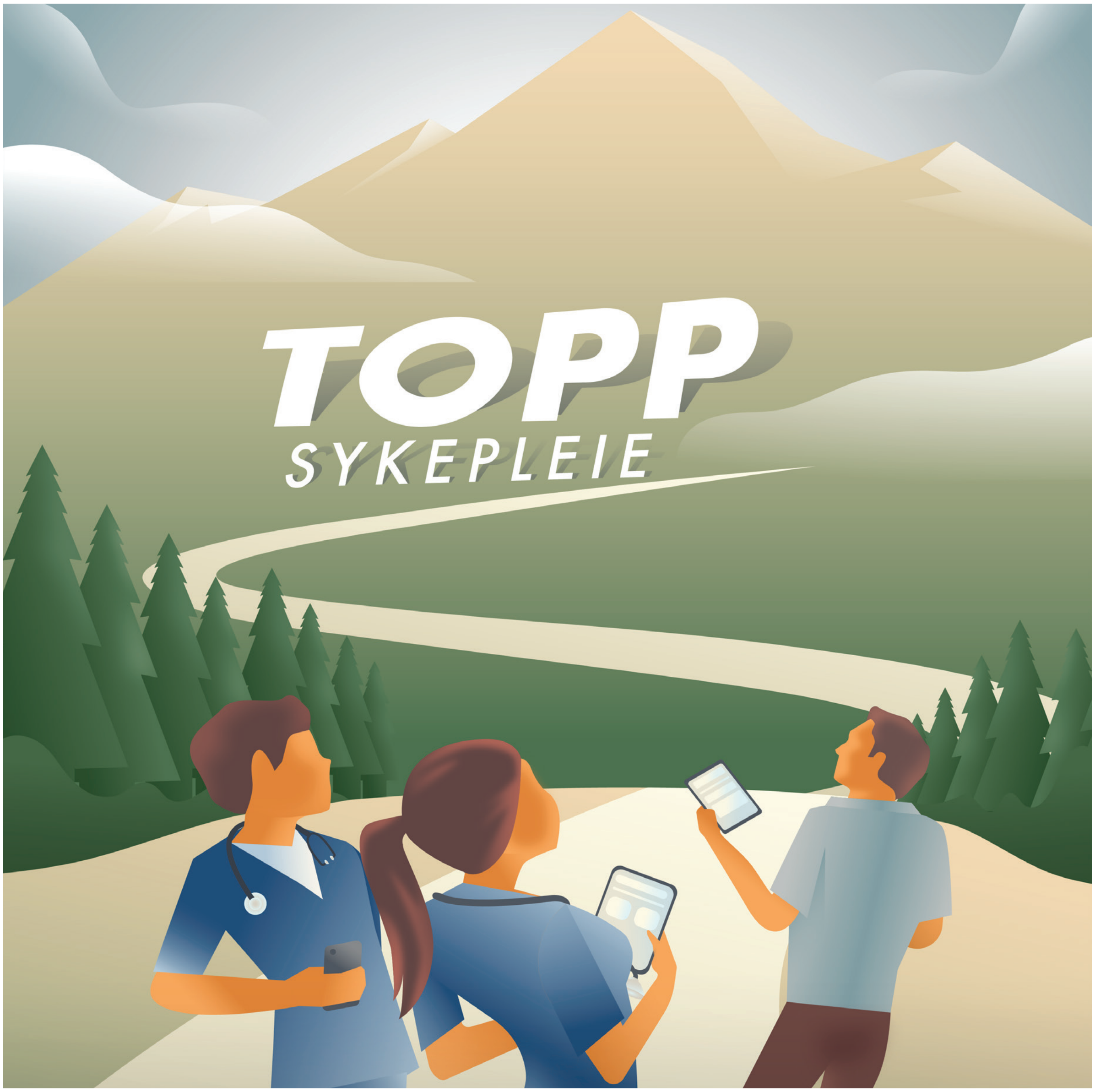

Hvis du har svarte ja på noen av disse spørsmålene er TOPP Sykepleie noe for deg!

Hva er TOPP Sykepleie (TOPP-S)?

TOPP Sykepleie er en applikasjon som er utviklet av og for veiledere, studenter og lærere for å gi støtte under praksisperioden. Med TOPP-S får du som veileder mulighet til å følge opp studenter ved hjelp av teknologi.

TOPP står for Teknologi Optimalisert Praksis Prosess.

Hvordan bruke TOPP-S applikasjonen?

Studenten bruker TOPP-S til å fylle ut daglige planer og rapporter basert på praksisperiodens kompetanseområder og læringsutbytter. Du som veileder får umiddelbart tilgang til informasjonen. Basert på denne informasjonen, samt daglig samarbeid med studenten, skriver eller sender du en taletilbakemelding til studenten via appen.

Hva får du som veileder ved å bruke TOPP-S?

- dokumentert oppfølging av studenten
- klarhet i hva som forventes av studenten under praksisperioden
- rask kontakt med læreren og støtte ved behov
- rask tilgang til informasjon
- økt veiledningskompetanse og kvalitet på din veiledning

Alt dette uten å bruke mer tid enn det du bruker per i dag.

Vil du være en av de første som tar i bruk TOPP-S?

Følg med på ldh.no/forskning/topp-sykepleie

Spørsmål?

Andréa Nes, prosjektleder, LDH:
andrea.nes@ldh.no

Jaroslav Zlamal, stipendiat, LDH:
jaroslav.zlamal@ldh.no

Lovisenberg diakonale høgskole

Diku

Les mer om prosjektet:
